# Evaluation of Nutrition and Performance Parameters in Division 1 Collegiate Athletes

**DOI:** 10.3390/nu16121896

**Published:** 2024-06-16

**Authors:** Marni E. Shoemaker, Nathan D. Dicks, Megan J. Northrup, Seth W. Daughters, Taylor N. Krings, Allison M. Barry

**Affiliations:** 1 School of Health and Consumer Sciences, South Dakota State University, Brookings, SD 57007, USA; megan.northrup@sdstate.edu (M.J.N.); seth.daughters@sdstate.edu (S.W.D.); taylor.krings@jacks.sdstate.edu (T.N.K.); allison.barry@sdstate.edu (A.M.B.); 2 Department of Health, Nutrition, and Exercise Sciences, North Dakota State University, Fargo, ND 58102, USA; nathan.dicks@ndsu.edu

**Keywords:** sports nutrition, maximal oxygen uptake, fat-free mass, aerobic capacity

## Abstract

Background: Testing and evaluating athletes is necessary and should include performance, body composition, and nutrition. The purpose of this study was to report assessments of dietary intake, $\dot{V}$O_2max_, and body composition in D1 collegiate athletes and examine relationships between these assessments. Methods: Dietary intake was assessed with 3-day recalls and compared to recommendations, and body composition was assessed via bioelectrical impedance analysis (BIA) (*n* = 48). $\dot{V}$O_2max_ was evaluated using a graded exercise test (GXT) with a verification bout (*n* = 35). Reliability between “true” $\dot{V}$O_2max_ and verification was determined. Correlations and regressions were performed. Results: Energy, carbohydrate, and micronutrient intake was lower than recommendations. Mean $\dot{V}$O_2max_ was 47.3 and 47.4 mL·kg^−1^·min^−1^ for GXT and verification, respectively. While correlations were apparent among dietary intake, $\dot{V}$O_2max_, and body composition, percent fat-free mass (%FFM) predicted 36% of $\dot{V}$O_2max_. Conclusions: Collegiate athletes are not meeting energy and carbohydrate recommendations and exceed fat recommendations. Vitamin D and magnesium were low in all sports, and iron and calcium were low in females. $\dot{V}$O_2max_ ranged from 35.6 to 63.0 mL·kg^−1^·min^−1^, with females below average and males meeting typical values for their designated sport. Assessing D1 athletes can provide guidance for sports dietitians, coaches, and strength and conditioning specialists to track and monitor nutrition in athletes.

## 1. Introduction

Assessing athletes is a necessary component for improving performance, including preventing injuries and illness. Testing components should include performance, body composition, and nutrition in order to provide guidance for maintaining health and injury prevention in athletes. The testing evaluation allows coaches, strength and conditioning specialists, and dietitians to understand where their athletes are currently at and what steps are needed to improve training and nutrition programs, and, in turn, develop an individual physiological profile for each athlete.

Collegiate athletes are often at risk of not meeting nutritional recommendations optimal for performance, indicating that nutrition is important to monitor in this population [1,2,3]. Adequate nutrition is essential for optimizing performance while decreasing the risk of developing injuries and illness [4]. In addition to energy intake (kcals) deficiencies, common themes observed in athletes include poor carbohydrate and vitamin D intake, as well as low protein, calcium, and iron intake, particularly in female athletes [1,2,3,4,5,6,7]. As these nutrients all play a key role in performance, determining and tracking athlete intakes is essential. Adequate calorie intake (kcals) is the foundation for the diet of athletes, allowing for appropriate energy production for the body to function and perform as needed. Additionally, meeting energy needs also allows for a sufficient intake of macronutrients and micronutrients, since it is difficult to meet those needs if overall calories are low [4]. Energy balance also plays a key role in body composition. Carbohydrates (endogenous and exogenous) are a highly preferred fuel source for training across a large range of exercise intensities that are both aerobic and anaerobic, making it an essential component of an athlete’s diet [8]. Carbohydrate recommendations for athletes are often provided based on the intensity of their activity and can be manipulated based on sex and sport [4,9]. Protein intake is necessary to support training adaptations, repair and rebuild tissue, and support protein turnover [10,11]. Additionally, many micronutrients such as vitamin D, calcium, and iron are all important for musculoskeletal health and performance [12,13,14,15,16]. Iron has a key role in red blood cell production, oxygen delivery, and electron transport during oxidative phosphorylation [17,18,19]. Vitamin D is not only necessary for the regulation of calcium and maintenance of bone health but is also thought to be influential on skeletal muscle, mainly due to the presence of vitamin D receptors (VDR) on skeletal muscle tissue [14,20]. Additionally, calcium has many functions important for athletic performance, including the maintenance of bone health, muscle contraction, and nerve conduction, making adequate intake essential [4]. Multiple nutrients are key components for skeletal muscle to improve athletic performance; thus, there should be an emphasis on dietary intake to ensure D1 athletes are consuming adequate amounts of macronutrients and micronutrients that are essential for sport performance, particularly with the specific needs for intermittent-based team and individual sports [4,21].

Maximal oxygen uptake ($\dot{V}$O_2max_) is considered the gold standard measurement for testing cardiorespiratory fitness and is commonly used to test athletes for evaluating training programs [22,23]. Sports such as volleyball, basketball, and wrestling have both aerobic (cardiorespiratory fitness) and anaerobic demands to not only provide energy for rapid and intermittent bouts of high-intensity activity but also sustain long periods of activity, indicating these athletes’ energy systems need to be efficient to maintain this level of performance [24,25,26,27]. Determining athletes’ $\dot{V}$O_2max_, particularly with the use of a verification bout, can provide an accurate assessment of aerobic capacity, which is useful for determining fitness levels and the effectiveness of training programs [28].

Body composition is another factor that influences performance and can be highly influenced by nutrition. Typically, in sports relying on power, such as many intermittent sports, a high proportion of skeletal muscle mass is advantageous for performance, so many athletes strive to gain or maintain fat-free mass (FFM) [4]. With the amount of interrelatedness between nutrition, performance, and body composition, it is important to evaluate all three in athletes and to examine relationships among these factors. Therefore, the purpose of this study was to report assessments of dietary intake, $\dot{V}$O_2max_, and body composition in D1 collegiate athletes and examine relationships between these assessments.

## 2. Materials and Methods

### 2.1. Study Design

This study reports descriptive values for dietary intake (energy, macronutrients, and micronutrients), $\dot{V}$O_2max_ performance values, and body composition assessments in male and female collegiate athletes of four different team and individual sports (women’s volleyball (WBB), women’s and men’s basketball (MBB), and men’s wrestling). Participants were assessed prior to the start of their respective competition season. 

### 2.2. Participants

A total of 48 collegiate athletes participated in this study, including female volleyball athletes (*n* = 13), WBB athletes (*n* = 11), MBB athletes (*n* = 14), and male wrestlers (*n* = 10). All participants were student–athletes at the same D1 university. All participants signed an informed consent approved by the university’s Institutional Review Board (IRB# 2308002-EXP) prior to participating in this study.

### 2.3. Surveys and Dietary Intake

Participants completed demographic, health history, and a Physical Activity Readiness Questionnaire (PARQ+) [29] prior to performing any other assessments. All participants were provided with a 3-day dietary recall approximately 7 days prior to the scheduled testing visit and were instructed to complete the record for days that were considered as typical as possible. A registered dietitian explained how to complete the recall by recording everything consumed over the 3 days. At the test visit, all participants were interviewed by a registered dietitian over their 3-day recall, where food intake was reviewed and follow-up questions were asked to the participants regarding additional intake, portion size (with visuals), brands, and preparation methods. The 3-day recalls were inputted into a nutrient analysis software program (Food Processor^®^, ESHA Research, Inc., Salem, OR, USA) and analyzed for energy, macronutrient, and micronutrient intake. The mean ± SD of energy intake (kcals·day^−1^), macronutrient intake (g·d^−1^), and specific micronutrients were analyzed for each day and averaged among the 3 days. 

### 2.4. Health, Anthropometrics, and Body Composition Measurements

Resting heart rate and blood pressure were measured after the participant remained in a seated position for 5 min. Blood pressure was taken manually with a sphygmomanometer and stethoscope by the same investigator. Heart rate was taken manually for 60 s. Height was measured using a stadiometer (Seca; Hamburg, Germany). Body mass, fat-free mass (FFM), fat mass (FM), and percent body fat (BF%) were assessed via bioelectrical impedance analysis (BIA) (InBody 570, Cerritos, CA, USA) [30]. Waist circumference was measured using a Gulick measurement tape (Baseline^®^ measurement tape with Gulick attachment, Fabrication Enterprises, White Plains, NY, USA) and recorded to the nearest 0.1 cm.

### 2.5. Maximal Oxygen Uptake ($\dot{V}$O_2max_)

We used a customized graded exercise test (GXT) followed by a subsequent exhaustive, square-wave bout to determine our criterion measure of “true” $\dot{V}$O_2max_. At the onset of this study, participants self-reported physical activity-rating (PA-R) on a scale from 0 to 15 [31]. The PA-R was utilized in a non-exercise regression equation using the participants’ sex, age, height, and weight to determine predicted maximal oxygen uptake ($\dot{V}$O_2max_) [32]. 

Predicted

(1) $$\dot{V}O_{2\max}\;(\mathrm{mL}\cdot {\mathrm{kg}}^{-1}\cdot \min^{-1})=(56.363)+(1.921\times \mathrm{PA}\text{-}R)-(0.381\times \mathrm{Age})-(0.754\times \mathrm{BMI})+(10.987\times \mathrm{Sex},\;\mathrm{male}=1,\;\mathrm{female}=2).$$

Researchers then estimated running speed to evoke $\dot{V}$O_2max_ (S_peak_) using the following metabolic equation [33]:

(2) $$S_{\mathrm{peak}}=(\dot{V}O_{2\max}-3.5)/0.2,$$

where S_peak_ is expressed in m·min^−1^, $\dot{V}$O_2max_ is relative to body mass (mL·kg^−1^·min^−1^), and 3.5 is the resting relative $\dot{V}$O_2_. The S_peak_ was projected to be achieved in the 10th stage of the protocol and was thus divided by 10 to calculate the change in each stage as well as the starting stage speed. Researchers implemented a safety speed cap of 238.71 m·min^−1^ on the treadmill (Woodway, PRO XL, Waukesha, WI, USA) and used an additional 1% grade to correspond with the projected metabolic gains associated with an added speed [34]. 

Participants were fitted with a facemask and expired through a two-way rebreathing valve (7450 V2, Hans Rudolph Inc., Shawnee, KS, USA) connected to a metabolic cart (Parvo Medics Inc., Sandy, UT, USA) to measure indirect calorimetry during the GXT. Telemetry HR (Polar Electro Inc., Lake Success, NY, USA) was recorded simultaneously, and all data were evaluated using 15 s averaging. Metabolic cart filter replacements and calibrations were performed between tests according to the manufacturer’s guidelines. 

Termination of the GXT occurred once the participant reached volitional fatigue and stopped the test by straddling the treadmill belt. The participant performed an active recovery for 3 min where the treadmill was set at 50% of the speed attained in the last stage. After recovery, the treadmill was set at a speed and grade, equaling two stages below the end stage of the GXT. Participants resumed running until exhaustion to confirm each participant’s “true” $\dot{V}$O_2max_ measured during the GXT. “True” $\dot{V}$O_2max_ was accepted as the highest values between the GXT and the square-wave bout when the agreement between trials was <3% [35]. 

### 2.6. Statistical Analysis

Assessments were described using means and standard deviations, and nutrition data were compared to recommendations based on Recommended Dietary Allowances (RDA) and for sport and sex. Initial steps were taken to ensure the normality of data before conducting statistical analysis. The normality of data was confirmed with visual representation from Q-Q plots. To examine mean differences between predicted and “true” $\dot{V}$O_2max_, dependent *t*-tests were performed. Pearson’s product-moment correlations were used to assess the association between energy intake, protein, carbohydrates, total fat, “true” $\dot{V}$O_2max_, %fat mass (FM), and %fat-free mass (FFM). A stepwise linear regressions was conducted using “true” $\dot{V}$O_2max_ as the dependent variable. The stepwise linear regression included energy intake, %FM, and %FFM independent variables. After the reporting of assessment data and relationships, a secondary analysis was performed posteriori including One-Way Between-Subjects ANOVAs conducted to assess sport differences in %FFM, %FM, “true” $\dot{V}$O_2max_, energy intake, protein, carbohydrates, and total fats. An alpha level of 0.05 was used to determine statistical significance. All statistical analyses were performed using SPSS 24 software (IMB Corp., Armonk, NY, USA).

## 3. Results

Table 1 reports means ± SD for demographics, anthropometrics, body composition, and $\dot{V}$O_2max_. Of the 48 athletes who completed dietary and body composition assessments, *n* = 35 completed the $\dot{V}$O_2max_ test due to injuries (volleyball, *n* = 8; WBB, *n* = 7; MBB, *n* = 13; wrestling, *n* = 7). 

### 3.1. Dietary Intake

Dietary intakes for energy, macronutrients, and micronutrients are displayed in Table 2 and compared to recommendations based on sex and sport (energy and macronutrients) [9,36] and RDAs (micronutrients) [37,38,39]. In general, reported energy and carbohydrate intakes were below recommendations for all sports, while protein intake was sufficient for MBB and wrestling but low for both female sports. Additionally, fat intake, including saturated fat, was high in all sports. Fiber intake was lower than recommendations for all sports and added sugars were high in all sports except wrestling (Table 2). For micronutrients, in general, B vitamins met the RDA for all sports, and male sports met or exceeded RDAs for calcium and iron but female sports did not. Almost every other micronutrient was low in comparison to RDAs for all sports, with particular concern for vitamin D and magnesium (Table 2).

### 3.2. $\dot{V}$O_2max_ Performance

**Figure 1:**
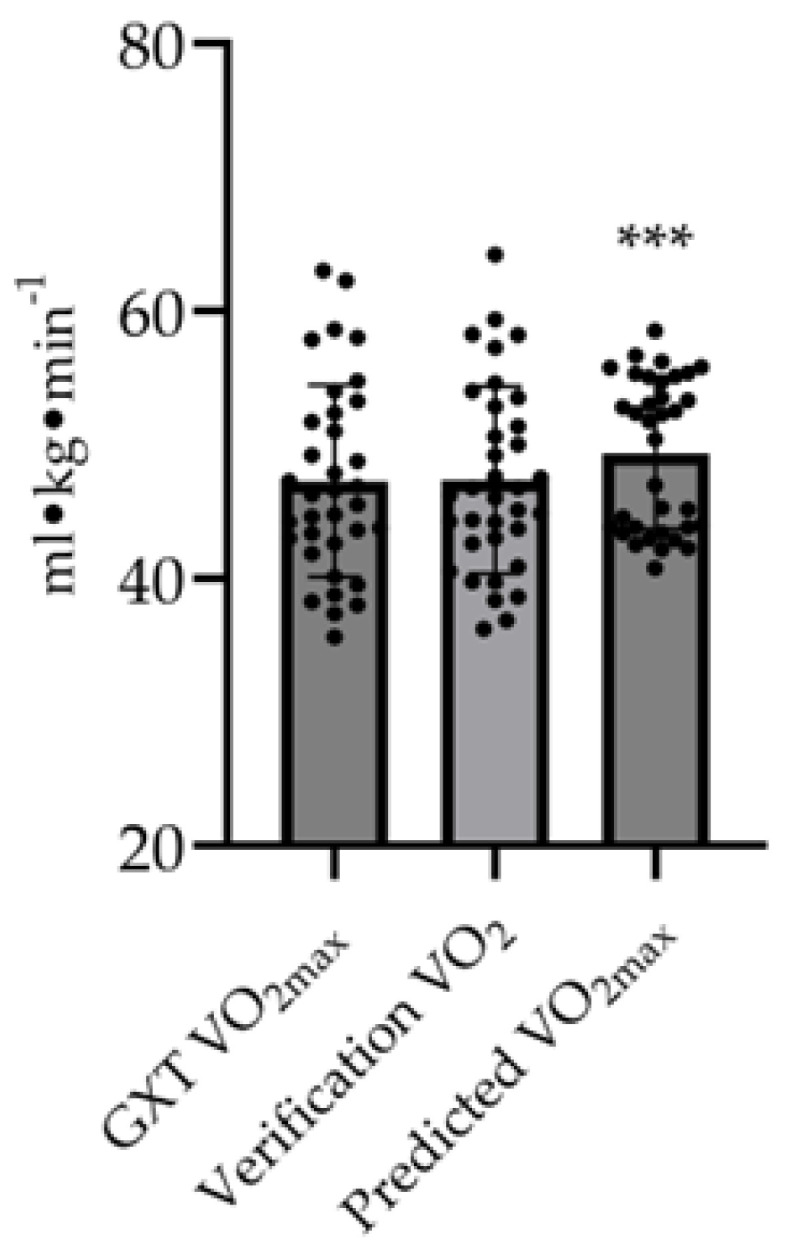
$\dot{V}$O_2max_ values from the GXT, square wave verification bout, and predicted value for D1 collegiate athletes. Predicted $\dot{V}$O_2max_ was significantly higher than “true” $\dot{V}$O_2max_ (*** *p* < 0.001), while there was high reliability between “true” $\dot{V}$O_2max_ and square wave verification bout (ICC = 0.97).

$\dot{V}$O_2max_ values from the GXT and square wave verification bout were 47.3 ± 7.2 and 47.4 ± 7.0 mL·kg^−1^·min^−1^, respectively (intraclass correlation coefficient (ICC) = 0.97, standard error of the mean = 1.2 mL·kg^−1^·min^−1^, coefficient of variation = 2.7%), with the highest value used to identify $\dot{V}$O_2max_. On average, the predicted $\dot{V}$O_2max_ was higher than the “true” $\dot{V}$O_2max_, which was statistically significant (*p* < 0.001) with a medium effect size (d = 0.38) (Figure 1). Refer to Table 3 for correlations.

Table 4 reports the stepwise linear regression with “true” $\dot{V}$O_2max_ as the dependent variable and energy intake, %FM, and %FFM as independent variables (R^2^ = 0.36). %FFM was predictive of “true” $\dot{V}$O_2max_ (F [1,33] = 18.89, R^2^ = 0.36, *p* < 0.001). %FFM predicted 36% of the variance in “true” $\dot{V}$O_2max_. 

### 3.3. Sport Differences

A One-Way Between-Subjects ANOVA was conducted to compare %FFM, %FM, “true” $\dot{V}$O_2max_, energy intake, protein, carbohydrates, and total fat to account for sport differences. It is important to note that due to Levene’s tests being considered non-significant for %FM, %FFM, and “true” $\dot{V}$O_2max_, homogeneity was not violated; however, there was a significant difference for energy intake, protein, carbohydrate, and total fat. The non-homogeneity for those variables are accounted for by the diverse eating behaviors within these athletes. There was a significant difference in %FFM based on sport (F(3,44) = 38.70, *p* < 0.001), with a partial η^2^ = 0.73. A post hoc analysis using a Bonferroni adjustment revealed that this was significantly lower in WBB vs. wrestling (*p* < 0.001), lower in WBB vs. MBB (*p* < 0.001), lower in volleyball vs. wrestling (*p* < 0.001), and lower in volleyball vs. MBB (*p* < 0.001). There was a significant difference in %FM based on sport (F(3,44) = 38.62, *p* < 0.001), with a partial η^2^ = 0.73. A post hoc analysis using a Bonferroni adjustment revealed %FM was significantly higher in WBB vs. wrestling (*p* < 0.001), higher in WBB vs. MBB (*p* < 0.001), higher in volleyball vs. wrestling (*p* < 0.001), and higher in volleyball vs. MBB (*p* < 0.001). There was a significant difference in “true” $\dot{V}$O_2max_ based on sport (F(3,31) = 18.78, *p* < 0.001), with a partial η^2^ = 0.65. A post hoc analysis using a Bonferroni adjustment revealed $\dot{V}$O_2max_ was significantly lower in WBB vs. wrestling (*p* < 0.01), lower in WBB vs. MBB (*p* < 0.001), lower in volleyball vs. wrestling (*p* < 0.01), lower in volleyball vs. MBB (*p* < 0.001), and lower in MBB vs. wrestling (*p* < 0.01). There was a significant difference in energy intake based on sport (F(3,44) = 8.38, *p* < 0.001), with a partial η^2^ = 0.36. A post hoc analysis using a Bonferroni adjustment revealed energy intake was significantly higher in MBB vs. WBB (*p* = 0.02), higher in MBB vs. volleyball (*p* < 0.001), and higher in MBB vs. wrestling (*p* = 0.027). There was a significant difference in protein based on sport (F(3,44) = 8.13, *p* < 0.001), with a partial η^2^ = 0.36. A post hoc analysis using a Bonferroni adjustment revealed protein intake was significantly lower in WBB vs. MBB (*p* = 0.013), lower in volleyball vs. MBB (*p* < 0.001), and lower in volleyball vs. wrestling (*p* = 0.032). There was a significant difference in carbohydrate based on sport (F(3,44) = 8.00, *p* < 0.001), with a partial η^2^ = 0.35. A post hoc analysis using a Bonferroni adjustment revealed carbohydrate intake was significantly higher in MBB vs. WBB (*p* = 0.002), higher in MBB vs. volleyball (*p* = 0.002), and higher in MBB vs. wrestling (*p* = 0.002). There was a significant difference in total fat based on sport (F(3,44) = 9.65, *p* < 0.001), with a partial η^2^ = 0.40. A post hoc analysis using a Bonferroni adjustment revealed higher fat intake in MBB vs. WBB (*p* = 0.001) and MBB vs. volleyball (*p* < 0.001).

## 4. Discussion

This study aimed to provide a descriptive assessment of dietary intake, $\dot{V}$O_2max_ performance, and body composition in D1 collegiate athletes of different sports. Additionally, this study investigated the relationship between nutrition and $\dot{V}$O_2max_ performance in D1 collegiate athletes and compared athletes’ dietary intake to nutritional recommendations. These assessments are important to provide context for other practitioners of team and individual sport athletes, particularly in settings where resources for nutritional support are limited. For nutrition, in general, collegiate athletes in all four sports are not meeting energy and carbohydrate intake recommendations and exceed fat and saturated fat recommendations. Females in both volleyball and WBB are low in protein intake, while male athletes are meeting protein requirements [4,9]. Additionally, micronutrient intake in general did not meet recommendations, with a particular concern for low vitamin D and magnesium consumption in all athletes and iron and calcium in females. Furthermore, there were differences in intakes based on sport, indicating potential trends that may be targets for team-based nutritional education. $\dot{V}$O_2max_ ranged from 35.6 to 63.0 mL·kg^−1^·min^−1^ across all athletes. The main contributor to $\dot{V}$O_2max_ performance was %FFM (Table 4), whereas dietary intakes in the current study were inconsequential to predicting $\dot{V}$O_2max_, potentially due to inadequate intakes of energy and carbohydrates in all sports. This finding was unexpected since meeting energy requirements is the foundation of an athlete’s diet for energy production, as well as the ability to achieve appropriate macronutrient intake; therefore, adequate intake would typically be an essential component for optimizing performance [4]. Additionally, carbohydrates are the primary fuel for high-intensity exercise, particularly for intermittent sports that have a combination of activity using both aerobic and anaerobic systems. Therefore, having a high aerobic capacity, as well as anaerobic ability, is needed throughout a game or match, which relies heavily on the contribution of aerobic ATP production, primarily fueled by carbohydrates, since it is the only fuel that can be utilized for both aerobic and anaerobic ATP production [8]. Since a $\dot{V}$O_2max_ test also relies on both aerobic and anaerobic domains, it was expected that energy and carbohydrates would have been influential to $\dot{V}$O_2max_ performance; however, this was not observed in the current study [8].

Energy intake depends on multiple factors including type of sport, sex, age, body mass, and height; however, unfortunately, many athletes are not meeting recommendations [2,3], which can severely impact the ability to train, perform, and recover optimally [9]. Previous reviews examining energy and macronutrient intakes in team sport athletes found similar patterns as the athletes in this study. Two separate reviews of professional or semi-professional team sport athletes reported that both male and females were also below recommendations for energy and carbohydrate intake and exceed consumption in fat intake [2,3], which was consistent with the findings for both male and female athletes in the present study. Typically, with intermittent-based sports, the aerobic system is greatly taxed, as well as the anaerobic and creatine phosphate system, indicating that carbohydrate intake should be a priority in these sports, being the primary fuel source for these types of activities [9,44]. Additionally, meeting energy intake recommendations to match expenditure is essential for athletes, as low intake can be associated with decreased performance, negative body composition effects such as reduced muscle mass, and an increased risk of injuries, with similar correlations seen in the present study among dietary intakes and body composition (Table 3) [9], making meeting energy and carbohydrate recommendations essential for athletes. 

Additionally, approximately 28.6% of the studies reviewed in a systematic review of indoor team sport athletes met recommendations for protein while 50% did not; however, there was no delineation of differences for sex or sport [2]. Low protein intake is common in female athletes [13,45], which was also confirmed in this study (Table 2). While not as important as a fuel source for performance, adequate protein is essential for muscle protein synthesis to tissue adaptations to training, recovery, and body composition changes, such as increased muscle mass [4,45]. Additionally, body composition testing can be influential to performance, depending on the type of athlete. Often, higher muscle mass with a low body fat percentage will help with intermittent and power-based athletes, such as the athletes tested in this study [4]. Additionally, better athletes typically have higher amounts of FFM; therefore, these athletes may also have a higher $\dot{V}$O_2max_, theoretically explaining why %FFM would be a large predictor of $\dot{V}$O_2max_ performance [46,47].

In addition to providing assessments of $\dot{V}$O_2max_, energy, and macronutrient intake, multiple micronutrients were also analyzed and reported, indicating a low intake in a majority of key nutrients for performance (Table 2). Several micronutrients have been highlighted as micronutrients of key interest for athletes, including iron, calcium, vitamin D, and antioxidants (including vitamins C, E, and beta-carotene) [4]. In general, all athletes had low intakes for these micronutrients, as well as many other vitamins and minerals analyzed in this study, with a specific concern for very low intakes of vitamin D and magnesium for all athletes, and iron and calcium in female athletes, which is an unfortunate common theme for female athletes [12,13,48,49]. 

There is growing evidence to support the role of vitamin D on skeletal muscle and performance, indicating that adequate intake is necessary for athletes. However, vitamin D consumption is typically low in most of the population, including athletes [1,50,51]. In addition to the importance of vitamin D for bone health in conjunction with calcium, vitamin D has a role in skeletal muscle function, potentially due to VDR expression on skeletal tissue influencing muscle cell regeneration [52,53]. Additionally, vitamin D concentrations have been reported to be associated with athletic performance [54,55,56]. With these being indoor athletes, as well as full-time students, it is highly likely that vitamin D created from exposure to UV radiation from sunlight is limited, which places a higher reliance on vitamin D intake. While vitamin D concentrations were not measured in this study, the combination of low vitamin D intake and low sun exposure with indoor athletes in addition to the location of the university suggests that concentrations may be low. Magnesium is emerging as a nutrient that could improve athletic performance due to its role in energy metabolism, muscle protein synthesis, and skeletal muscle contraction [57,58,59]. While few human studies have been performed to link magnesium intake with athletic performance, preliminary evidence supports positive associations between magnesium levels and markers of performance such as strength and power [59,60]. Iron is essential for multiple processes within the body that influence performance, including oxygen delivery and utilization, energy metabolism, and red blood cell production [17,18,19], indicating that low concentrations could have an adverse impact on performance. For example, the presence of iron deficiency was related to lower $\dot{V}$O_2max_ in female rowers compared to those with sufficient iron status [61]. Additionally, iron supplementation has been reported to improve tests of aerobic capacity (4k time trial and $\dot{V}$O_2max_ performance) in endurance athletes [62,63]. Since low iron intake is common in female athletes and is particularly concerning with the greater requirements for this population, this becomes a micronutrient necessary to monitor in athletes [4]. Similarly, to iron, females typically do not meet RDAs for calcium, which becomes even more important for athletes due to the role calcium has in bone health and skeletal muscle function [4,64]. With the great importance that these micronutrients have for performance, educating athletes on consuming food sources rich in these micronutrients, as well as meeting energy and macronutrient recommendations, is essential for optimizing performance and reducing the risk of injury and illness.

Maximal oxygen consumption is utilized as a test of functional capacity of the cardiovascular and respiratory systems, along with oxygen utilization of skeletal muscle, indicating that this assessment is considered the criterion measurement of cardiorespiratory fitness [33]. Sports such as volleyball, basketball, and wrestling require an interplay of the aerobic (cardiorespiratory fitness) and anaerobic capacity, meaning an efficient energy system is optimal to sustain intermittent and sustained activity [24,25]. Thus, assessing $\dot{V}$O_2max_ in collegiate teams and individual athletes can provide coaches with a determinant of their athletes’ physical capacity and how it changes based on training protocols throughout the season. The use of a verification bout in lieu of the traditional $\dot{V}$O_2max_ test criteria has been a promising technique to confirm that an actual $\dot{V}$O_2max_ has been reached [28]. Few studies have examined the confirmation of attaining $\dot{V}$O_2max_ with a verification bout in college athletes, making this a novel technique in this population [28]. With the high reliability between the “true” $\dot{V}$O_2max_ and $\dot{V}$O_2max_ from the verification bout in the present study (Figure 1), this would be an effective method for testing collegiate athletes to ensure the accuracy of the test. 

Intermittent, power-based sports such as basketball and volleyball require a developed aerobic capacity to be able to sustain the frequent bouts of activity and have better tolerance to fatigue caused from multiple high-intensity spurts [25,65,66]. Therefore, a certain level of cardiorespiratory fitness should be maintained in these types of athletes. As expected, the male sports had a higher $\dot{V}$O_2max_ than females. Previously, $\dot{V}$O_2max_ ranged from 48 to 51 mL·kg^−1^·min^−1^ in male basketball players in national leagues and from 50 to 58 mL·kg^−1^·min^−1^ in collegiate male basketball players [27], which was slightly higher than the 48.8 mL·kg^−1^·min^−1^ reported in this study (Table 1). Fewer studies examined female basketball players; however, one previous study in female D1 collegiate basketball players reported a mean $\dot{V}$O_2max_ value of 47.3 mL·kg^−1^·min^−1^ [67], which was higher than the reported value of 41.8 mL·kg^−1^·min^−1^ in the present study (Table 1). There are very few studies examining $\dot{V}$O_2max_ in female volleyball players; however, compared to the descriptive data for athletes, the $\dot{V}$O_2max_ of 40.7 mL·kg^−1^·min^−1^ reported for the volleyball athletes would have been considered below average in comparison to other athletes and other previously reported values of female volleyball players [68,69]. While volleyball is a high-intensity, intermittent sport and relies greatly on anaerobic capacity, the ability to maintain a level of intensity throughout a match, as well as the ability to recover and reduce fatigue to perform over the course of five matches, places some reliance on the oxidative system, indicating that a level of cardiorespiratory fitness and aerobic capacity would be important for performance in this sport [70]. High-performing wrestlers were reported to have $\dot{V}$O_2max_ values between 52 and 63 mL·kg^−1^·min^−1^ [26], which aligns with the wrestlers in the present study.

### Strengths and Limitations

The strengths of this study emphasize a well-rounded assessment including in-depth nutrition information of multiple different sports within a D1 university setting, including an analysis of dietary intake and $\dot{V}$O_2max_ testing in athletes. Very few studies provide in-depth dietary analysis of different D1 sports, particularly including in-depth interviews with each athlete, reports of macronutrient and micronutrient intake, and comparisons to recommendations (relative recommendations (g·kg^−1^) per sport for macronutrients and RDAs for micronutrients). Additionally, this study provides an assessment of $\dot{V}$O_2max_ with a protocol using a verification bout to ensure that true $\dot{V}$O_2max_ was reached. This can provide helpful data for other coaches, strength and conditioning specialists, and sports dietitians to compare with. However, there are some limitations to this study. This study aimed to show the assessment data of different sports at the D1 collegiate level with limited resources for nutritional support. All athletes came from the same D1 university, leading to each sport having a small sample size. However, we were able to achieve more than 70% participation for each team’s starting roster, which is fairly difficult in a collegiate athletic setting. With this sample size, there are certain limitations for regression analysis, particularly with the influence of sex on the analysis. With *n* = 35 completing the $\dot{V}$O_2max_ assessment (*n* = 20 males, *n* = 15 females), having three independent variables within the model meets the rule of 10 participants for every independent variable added into the model; however, there still may be an influence of sex on these results. There is also the potential for error associated with dietary recalls as they rely on self-reporting and portion size estimation. While we tried to limit error as much as possible by providing instructions and interviewing each athlete over their completed recall, along with visuals for portions sizes, this method is still susceptible to error. Additionally, the assessment of body composition was performed via BIA which has limitations, particularly associated with the hydration status of the athletes. All athletes were instructed to hydrate prior to the test and were asked about fluid consumption when they arrived at the laboratory to reduce the risk of athletes arriving dehydrated and adversely affecting the test. However, it is acknowledged that this method has limitations, but it was deemed the most appropriate method due to the time burden to athletes and the cost of equipment.

## 5. Conclusions

This study aimed to report nutritional intake, $\dot{V}$O_2max_ performance, and body composition in D1 collegiate athletes in four team and individual sports (volleyball, WBB, MBB, and wrestling), to provide a well-rounded assessment of athletes at a university with little nutritional support and to provide better context for sports dietitians, coaches, and strength and conditioning specialists to focus on within their sports. In general, dietary intake did not meet recommendations, particularly for energy, carbohydrates, and micronutrients, indicating the need for additional support of a registered dietitian within the interdisciplinary team. Athletes should focus on improving their energy and carbohydrate intake, as well as choosing nutrient-dense foods to not only support performance but meet micronutrient needs. $\dot{V}$O_2max_ performance ranged from 35.6 to 63.0 mL·kg^−1^·min^−1^, with female athletes considered below average and male athletes meeting typical values for their designated sport. $\dot{V}$O_2max_ performance in these athletes was adequate, even with nutritional intake below recommendations. However, by improving dietary intake to meet requirements per sport and sex, performance could be optimized. Additionally, since %FFM was predictive of $\dot{V}$O_2max_ performance, improving body composition, as well as monitoring nutrition, should be essential components in addition to their training programs for collegiate athletes.

## Figures and Tables

**Table 1:** Demographics, anthropometrics, body composition, and $\dot{V}$O_2max_ performance in D1 collegiate volleyball, women’s basketball, men’s basketball, and wrestling athletes.

	Total	Volleyball	Women’s Basketball	Men’s Basketball	Wrestling
	(*n* = 48)	(*n* = 13)	(*n* = 11)	(*n* = 14)	(*n* = 10)
Age (years)	19.6 ± 1.5	19.1 ± 1.0	19.8 ± 1.6	19.9 ± 1.9	19.9 ± 1.6
Height (cm)	182.2 ± 11.6	177.8 ± 8.3	179.5 ± 6.4	194.6 ± 9.0	173.8 ± 9.5
Body Mass (kg)	82.5 ± 12.2	74.8 ± 6.8	80.7 ± 8.3	93.2 ± 11.9	79.8 ± 12.6
Fat Mass (kg)	14.5 ± 5.3	18.0 ± 3.7	18.2 ± 3.7	10.7 ± 3.7	11.1 ± 4.8
Fat-Free Mass (kg)	68.1 ± 12.7	56.8 ± 5.0	62.5 ± 6.1	82.5 ± 9.1	68.7 ± 9.8
Fat Mass Percent (%)	17.7 ± 6.5	23.5 ± 3.7	22.4 ± 3.5	11.3 ± 2.6	13.7 ± 4.3
Fat-Free Mass Percent (%)	82.3 ± 6.5	76.5 ± 3.7	77.6 ± 3.5	88.7 ± 2.6	86.3 ± 4.3
Waist Circumference (cm)	80.9 ± 6.1	77.2 ± 4.0	80.7 ± 5.4	83.8 ± 6.9	81.9 ± 7.3
Resting Heart Rate (bpm)	70 ± 13.0	73.8 ± 10.1	70.0 ± 11.9	76.4 ± 10.9	56.0 ± 10.4
Systolic Blood Pressure (mmHg)	120.0 ± 4.4	118.8 ± 4.4	121.5 ± 5.8	121.6 ± 2.5	117.8 ± 4.2
Diastolic Blood Pressure (mmHg)	77.3 ± 8.1	76.4 ± 5.7	77.3 ± 5.8	77.4 ± 3.3	78.5 ± 15.7
$\dot{V}$O_2max_ (mL·kg^−1^·min^−1^) *	47.3 ± 7.2	40.8 ± 3.1	41.8 ± 3.1	48.8 ± 5.1	57.5 ± 4.2

* *n* = 35 athletes completed the $\dot{V}$O_2_max test (volleyball, *n* = 8; WBB, *n* = 7; MBB, *n* = 13; wrestling, *n* = 7).

**Table 2:** Energy, macronutrient, and micronutrient intake and comparisons to recommendations for D1 collegiate volleyball, women’s basketball, men’s basketball, and wrestling athletes.

	All Athletes	Volleyball		Women’s Basketball		Men’s Basketball		Wrestling	
**Dietary Intake**	**Intake**	**Intake**	**Recommendation**	**Intake**	**Recommendation**	**Intake**	**Recommendation**	**Intake**	**Recommendation**
Energy (kcals)	2650 ± 1233	1979 ± 396	2550–2750 ^a^	2165 ± 336	3100–3300 ^a^	3934 ± 1409	4100–4300 ^a^	2500 ± 720	3500–3700 ^a^
Carbohydrates (g)	291 ± 142	241 ± 63	375–450 ^b^	237 ± 54	400–565 ^b^	435 ± 167	550–650 ^b^	232 ± 69	480–550 ^b^
Protein (g)	130 ± 65	83 ± 16	135–150 ^c^	107 ± 45	145–161 ^c^	173 ± 66	168–186 ^c^	149 ± 72	143–160 ^c^
Fat (g)	114 ± 52	79 ± 16	70–75 ^d^	92 ± 21	75–80 ^d^	166 ± 59	90–95 ^d^	120 ± 35	75–80 ^d^
Saturated Fat (g)	37 ± 20	25 ± 7	15–18 ^e^	29 ± 7	18–21 ^e^	56 ± 24	23–28 ^e^	36 ± 13	20–24 ^e^
Trans Fat (g) ^f^	0.7 ± 0.7	0.4 ± 0.5	<1% kcals	0.5 ± 0.3	<l% kcals	1.2 ± 0.9	<1% kcals	0.8 ± 0.7	<1% kcals
Fiber (g) ^g^	19 ± 15	18 ± 6	25–26 ^g^	12 ± 5	25–26 ^g^	18 ± 18	38 ^g^	22 ± 12	38 ^g^
Added Sugars (g) ^h^	43 ± 30	51 ± 27	25 ^g^	28 ± 19	25 ^g^	54 ± 31	36 ^g^	26 ± 18	36 ^g^
Sodium (mg) ^i^	4433 ± 2370	2924 ± 776	3800	3571 ± 1010	3800	7369 ± 1943	3800	3873 ± 1499	3800
**Micronutrients**	**Intake**	**Intake**	**% RDA**	**Intake**	**% RDA**	**Intake**	**% RDA**	**Intake**	**% RDA**
Vitamin A (µg)	727.8 ± 584.3	884.2 ± 815.5	126%	534.0 ± 490.7	76%	836.0 ± 524.7	93%	585.7 ± 330.4	65%
Vitamin B1 (mg)	1.0 ± 0.8	0.7 ± 0.4	63%	0.8 ± 0.6	73%	1.3 ± 1.3	108%	1.0 ± 0.4	83%
Vitamin B2 (mg)	2.0 ± 2.2	1.1 ± 0.5	100%	1.5 ± 1.1	136%	2.1 ± 1.7	162%	2.3 ± 1.3	177%
Vitamin B3 (mg)	21.6 ± 16.4	15.1 ± 6.5	108%	20.8 ± 17.1	149%	21.3 ± 16.2	133%	33.0 ± 20.0	206%
Vitamin B6 (mg)	2.1 ± 1.7	1.4 ± 1.0	108%	2.1 ± 1.5	162%	2.1 ± 1.9	162%	2.7 ± 1.5	208%
Vitamin B12 (µg)	5.5 ± 5.0	3.2 ± 2.3	133%	5.2 ± 4.7	217%	5.2 ± 4.0	217%	8.5 ± 7.1	354%
Vitamin C (mg)	67.7 ± 38.4	66.5 ± 35.3	89%	57.0 ± 35.1	76%	60.2 ± 38.5	67%	81.1 ± 46.6	90%
Vitamin D (µg)	4.2 ± 5.4	3.3 ± 2.7	22%	2.3 ± 2.0	15.3%	5.0 ± 6.9	33%	6.1 ± 7.6	41%
Calcium (mg)	1080.8 ± 754.5	759.7 ± 367.5	76%	726.6 ± 215.2	73%	1247.2 ± 807.5	124%	1247.2 ± 807.5	125%
Iron (mg)	17.0 ± 18.2	12.6 ± 4.1	70%	11.2 ± 5.3	62%	18.1 ± 8.3	226%	15.4 ± 6.9	193%
Magnesium (mg)	197.2 ± 10.47	184.1 ± 84.2	59%	165.0 ± 99.2	53%	198.5 ± 104.0	50%	241.1 ± 129.8	60%
Potassium (mg)	2508.1 ± 1374.9	1876.5 ± 625.5	72%	2079.6 ± 1087.7	80%	2691.6 ± 1407.1	79%	3001.7 ± 1393.1	88%
Zinc (mg)	8.4 ± 6.5	6.3 ± 3.5	79%	7.3 ± 8.1	91%	9.1 ± 5.3	83%	12.6 ± 7.7	115%
Omega 3 (g)	0.8 ± 0.8	0.6 ± 0.3	55%	0.9 ± 0.5	82%	0.7 ± 0.5	44%	0.7 ± 0.3	44%
Omega 6 (g)	7.4 ±4.7	7.5 ± 4.1	63%	7.9 ± 6.2	66%	7.3 ± 4.9	43%	6.9 ± 3.8	41%

^a^ Recommendations for energy were calculated using the Cunningham equation [36]: ((500 + 22 × FFM) × Activity Factor (AF), where AF: volleyball = 1.5, women’s basketball (WBB) = 1.7, men’s basketball (MBB) = 1.8, and wrestling = 1.8), Fat-free mass (FFM, kg). ^b^ Carbohydrates: volleyball = 5–6 g·kg^−1^, WBB = 5–7 g·kg^−1^, MBB = 6–7 g·kg^−1^, wrestling = 6–7 g·kg^−1^ [9]. ^c^ Protein: volleyball = 1.8–2.0 g·kg^−1^, WBB = 1.8–2.0 g·kg^−1^, MBB = 1.8–2.0 g·kg^−1^, wrestling = 1.8–2.0 g·kg^−1^ [9]. ^d^ 1 g·kg^−1^ fat (±5) [9]. ^e^ Saturated fat recommendation from the American Heart Association (AHA) [40]. ^f^ Trans-fat recommendation of <1% kcals from the World Health Organization [41]. ^g^ Fiber recommendations [42]. ^h^ Added sugars recommendation from the AHA [43]. ^i^ Sodium recommendations of 3.8 g salt per day (1.5 g of sodium and 2.3 g of chloride) from the Institute of Medicine [38]. RDA; Recommended Dietary Allowance.

**Table 3:** Correlations among dietary intakes, “true” and predicted $\dot{V}$O_2max_, and body composition in D1 collegiate athletes.

Variable	1	2	3	4	5	6	7	8
1. Energy	-	0.73 **	0.95 **	0.95 **	0.19	0.16	−0.56 **	0.56 **
2. Protein		-	0.58 **	0.72 **	0.35 *	0.06	−0.59 **	−0.59 **
3. Carbohydrates			-	0.83 **	0.08	0.21	−0.49 **	−0.49 **
4. Total Fat				-	0.29	0.15	−0.59 **	0.59 **
5. “True” $\dot{V}$O_2max_					-	0.69 **	−0.65 **	0.65 **
6. Predicted $\dot{V}$O_2max_						-	−0.33 *	0.33 *
7. % Fat Mass							-	−1.0 **
8. % Fat-free Mass								-

* *p* < 0.05, ** *p* < 0.01.

**Table 4:** Stepwise regression model.

Variable	B	SEB	ß
Constant	−12.900	13.899	
% FFM	0.721 ***	0.166	0.603

*** *p* < 0.001. Note: R^2^ = 0.364; adjusted R^2^ = 0.345.

## Data Availability

The data presented in this study are available on request from the principal investigator.
